# Emotional labor, job satisfaction, and retention among home care workers in Taiwan: a comprehensive analysis

**DOI:** 10.3389/fpsyg.2025.1545955

**Published:** 2025-03-31

**Authors:** Tung-Sheng Kuo, Li-Chuan Chu, Chia-Lung Shih, Ya-Ching Li, Pi-Lien Kao

**Affiliations:** ^1^Department of Business Administration, Nanhua University, Chiayi County, Taiwan; ^2^Clinical Research Center, Ditmanson Medical Foundation Chia-Yi Christian Hospital, Chiayi, Taiwan; ^3^Home Care Nursing Agency, Ditmanson Medical Foundation Chia-Yi Christian Hospital, Chiayi, Taiwan; ^4^Discharge Service Section, Ditmanson Medical Foundation Chia-Yi Christian Hospital, Chiayi, Taiwan; ^5^General Affairs Office, Ditmanson Medical Foundation Chia-Yi Christian Hospital, Chiayi, Taiwan

**Keywords:** emotional labor, job satisfaction, willingness to stay, home-care worker, retention intention

## Abstract

**Objective:**

This study aims to investigate the relationships between emotional labor, job satisfaction, and retention among home care workers in Taiwan.

**Materials and methods:**

This study is a cross-sectional study design. Data were randomly collected from home care workers in Taiwan’s Chiayi region through questionnaires. We included 365 participants, primarily female (80.3%), with ages ranging from 18 to 60 years old. The questionnaires assessed emotional labor, job satisfaction, and retention intention. Data analysis was conducted using IBM SPSS Statistics software (version 28.0) and included descriptive statistics, factor analysis, reliability analysis, t-tests, one-way ANOVA, Pearson correlation coefficients, K-means cluster analysis, and path analysis.

**Results:**

The study included 365 participants, with females comprising the majority (80.3%). Emotional labor exhibits a significant positive relationship with job satisfaction (β = 0.157, *p* < 0.01) and willingness to stay (β = 0.115, *p* < 0.05). However, job satisfaction shows no significant relationship with willingness to stay (β = 0.48, *p* > 0.05). The participants were arbitrarily classified into two clusters based on cluster analysis. Cluster 1 exhibited more genuine emotional expressions (deep acting) during work, demonstrated higher job satisfaction, and showed a greater willingness to stay.

**Conclusion:**

The study provides insights into the relationships between emotional labor, job satisfaction, and retention among home care workers in Taiwan. The findings aim to offer valuable insights for managers in hospitals and long-term care facilities to enhance their recruitment strategies and personnel management practices.

## 1 Introduction

With 16.91% of its population aged 65 and above as of January 2022, Taiwan is experiencing a dramatic demographic shift toward becoming a super-aged society (Chiu, 2022). This shift in demographics has driven a surge in demand for long-term care services, with home care being the predominant choice—accounting for 94.2% of all long-term care services delivered in 2017 (Chiu, 2022). The combination of an aging society, lower fertility rates, and evolving household dynamics has put significant strain on traditional care systems where families are the primary caregivers (Hsu et al., 2018). In response to these demographic shifts, Taiwan is developing a comprehensive long-term care framework modeled after Japan’s successful Community Inclusive Care System (Hsieh et al., 2016). Taiwan’s healthcare system is evolving from treating acute conditions to managing long-term illnesses, requiring collaboration across multiple healthcare disciplines to deliver comprehensive home care (Hsu et al., 2018).

In response to its aging demographic, Taiwan has launched major long-term care (LTC) initiatives. A key development was the 10-Year Long-Term Care Plan 2.0 in 2017, which enhanced the existing tax-based system to deliver wider access and more extensive LTC support services (Yeh, 2019). This policy reform has significantly expanded both the number of healthcare providers and the reach of long-term care services (Chen and Fu, 2020). Despite these initiatives, significant challenges persist, including insufficient support ratios between working-age and elderly populations, shortages in qualified healthcare professionals, and gaps in LTC education and training programs (Wang and Tsay, 2012). The government is taking a coordinated approach to enhance LTC by unifying ministerial oversight, streamlining policies, and implementing graduated subsidy programs alongside diverse service delivery options (Huang and Chia-Ying, 2019).

Taiwan’s aging population has driven increased demand for home care services, yet the sector continues to face significant workforce challenges. Although government initiatives in 2018 introduced new payment structures that increased the home care workforce by 31.8% (Wu et al., 2021), overall employment in the sector remains at just 53%. Key factors that affect staff retention include how connected employees feel to their workplace, their loyalty to the organization, and whether they feel secure expressing themselves at work (Chang et al., 2023). Research shows that staff retention is most directly influenced by workplace satisfaction, effective leadership, and a culture of respect, while compensation plays a secondary role in retention decisions (Hsu and Shih, 2023).

Home care workers often experience significant physical and emotional stress in their roles. This contributes to a relatively low retention rate among home care workers in Taiwan. To address the increasing need for home care services in Taiwan, this study examines key factors influencing home care worker retention, particularly emotional labor and job satisfaction. The findings aim to provide valuable insights for institutions to enhance retention rates and meet the growing demand.

## 2 Literature overview

### 2.1 Emotional labor

Emotional labor involves managing and adjusting one’s emotions and emotional expressions to conform to workplace expectations and standards (Ashforth and Humphrey, 1993; Grandey and Sayre, 2019). Emotional labor includes surface acting, deep acting, or expressing genuine emotions (Ashforth and Humphrey, 1993). Although emotional labor can improve job performance and client satisfaction, it can also create emotional conflicts and feelings of disconnection from one’s authentic self (Ashforth and Humphrey, 1993; Bono and Vey, 2005). When caregivers genuinely modify their emotional state to match workplace expectations (deep acting), it tends to enhance their job performance. In contrast, merely pretending to display appropriate emotions (surface acting) can lead to stress and reduced mental well-being (Grandey and Sayre, 2019).

Managing and displaying appropriate emotions at work, known as emotional labor, has become a key determinant of workplace effectiveness (Guoyu et al., 2024). Studies indicate that when employees genuinely engage with their emotions (deep acting) or express natural feelings at work, it benefits everyone involved—workers experience greater job satisfaction, organizations see improved performance, and customers receive better service (Humphrey et al., 2015). Organizations that place a higher emphasis on emotional management tend to achieve better client relationships and enhanced operational outcomes (Meier et al., 2006). Nevertheless, the demands of emotional labor can lead to mental fatigue and professional exhaustion when not properly managed (Schiopu, 2014). Understanding how emotional labor functions enables organizations to better manage its effects and improve workplace effectiveness (Schiopu, 2014).

Adelmann (1989) found that emotionally intensive work can negatively affect employees (Adelmann, 1989). Those experiencing high emotional arousal face greater emotional dissonance, leading to poorer job performance and satisfaction compared to employees with lower emotional arousal. Studies suggest that emotional labor in caregiving professions can contribute to burnout and decreased job satisfaction (Roh et al., 2016; Jeung et al., 2018). In contrast, deep acting can enhance positive emotions, improve overall job performance, and increase one’s sense of personal achievement (Brotheridge and Grandey, 2002). Zapf (2002) argues that employees should view emotional management and emotional labor as essential components of their job, rather than as additional tasks separate from their primary responsibilities (Zapf, 2002).

### 2.2 Job satisfaction

Hoppock (1935) pioneered the concept of job satisfaction, defining it as employees’ subjective contentment with their work environment, encompassing both physiological and psychological aspects (Hoppock, 1935). This initial definition viewed job satisfaction through a singular lens. However, contemporary researchers argue that job satisfaction is multifaceted, comprising various elements such as the nature of work, career advancement prospects, remuneration, relationships with supervisors, and interactions with colleagues (Prosen and Piskar, 2015).

Herzberg et al. (1959) introduced the Two-Factor Theory, also called the Motivation-Hygiene Theory. This theory posits that job satisfaction stems from two distinct types of factors: motivators (or satisfiers) and hygiene factors (Herzberg et al., 1959). Motivators are intrinsic factors such as achievement, responsibility, advancement, and personal growth. In contrast, hygiene factors relate to the job’s context rather than its content.

The most widely accepted definition of job satisfaction describes it as a positive emotional state resulting from the fulfillment of one’s expectations and needs at work (Kader et al., 2021). Weiss et al. (1967) further categorized job satisfaction into two distinct types: intrinsic and extrinsic satisfaction (Weiss et al., 1967). Intrinsic satisfaction stems directly from the nature of the job itself, encompassing factors such as self-esteem and a sense of accomplishment. In contrast, extrinsic satisfaction arises from factors not directly related to the job’s core responsibilities, including salary and relationships with colleagues.

### 2.3 Retention intention

Retention intention refers to an employee’s conscious and deliberate intention to remain with their organization, indicating their commitment to their current position (Tett and Meyer, 1993). Porter et al. (1974) proposed that willingness to stay comprises three components: 1. Desire to Stay—enjoying one’s current position and wanting to continue in it; 2. Tendency to Stay—having no intention to quit and not seeking other job opportunities; and 3. Actual Retention—currently remaining in the same position (Porter et al., 1974). Employee retention is a key indicator of organizational health. Turnover can be distressing for both the organization and individuals. For employees, leaving a job can disrupt their social life, severing relationships with team members, supervisors, subordinates, and informal groups. For organizations, departing employees take with them the investment in training, knowledge capital, and even relationship capital with external stakeholders—costs the organization must absorb. Consequently, employers recognize the critical importance of retaining talent (Ghosh et al., 2013).

Walker (2001) identified seven factors contributing to employee retention: performance recognition and rewards, challenging work, opportunities for learning, positive collegial relationships, acknowledgment of capabilities and contributions, healthy work-life balance, and effective communication (Walker, 2001). Ribelin (2003) identified several key factors influencing the retention of talented employees (Ribelin, 2003). These include job satisfaction, corporate image, the company’s future development prospects, corporate culture, job content, sense of achievement, workload, and promotion opportunities. Additionally, training programs, leadership style, salary and benefits, camaraderie among colleagues, work environment, and work-life balance play crucial roles in employee retention. Shahid (2018) emphasized that organizational trust is a crucial factor in employees’ decisions to remain with a company and in the trust, they develop towards their employer (Shahid, 2018).

### 2.4 Relationship between variables

#### 2.4.1 Relationship between emotional labor and job satisfaction

Research by Kammeyer-Mueller et al. (2013) reveals that surface acting negatively correlates with job satisfaction, whereas deep acting shows a positive correlation (Kammeyer-Mueller et al., 2013). Grandey (2000) highlighted that employees who practice deep acting are less prone to emotional distress and typically achieve higher levels of customer satisfaction (Grandey, 2000). Deep acting enhances positive emotions and overall job performance, fostering a sense of personal accomplishment (Brotheridge and Grandey, 2002).

Diefendorff et al. (2005) categorized emotional display rules as either positive (active) or negative (passive) (Diefendorff et al., 2005). Their research indicates that positive display rules are a significant predictor of deep acting, while negative display rules show a positive correlation with surface acting. Several studies have demonstrated a positive correlation between certain emotional labor strategies and job satisfaction (Lee and Ok, 2012; Kaur and Malodia, 2017; Kim et al., 2017). According to the current evidence, emotional labor is associated with job satisfaction.

Research by Kammeyer-Mueller et al. (2013) reveals a negative correlation between surface acting and job satisfaction, while demonstrating a positive correlation between deep acting and job satisfaction (Kammeyer-Mueller et al., 2013). Grandey (2000) highlighted that employees who practice deep acting are less prone to emotional distress and typically achieve higher levels of customer satisfaction (Grandey, 2000). Deep acting enhances positive emotions, improves overall job performance, and fosters a sense of personal accomplishment (Brotheridge and Grandey, 2002). Diefendorff et al. (2005) classified emotional display rules as either positive or negative (Diefendorff et al., 2005). Their research indicates that positive display rules strongly predict deep acting, while negative display rules show a positive correlation with surface acting. Several studies confirm a positive relationship between emotional labor strategies and job satisfaction (Lee and Ok, 2012; Kaur and Malodia, 2017; Kim et al., 2017). In conclusion, the literature demonstrates a clear influence of emotional labor on job satisfaction.

#### 2.4.2 The relationship between job satisfaction and retention intention

Radford and Meissner (2017) found that employees in long-term care settings—both institutional and community-based—with higher job satisfaction are more likely to stay in their positions (Radford and Meissner, 2017). Coetzee and Stoltz (2015) found that employees’ willingness to stay is high when they align with the organization’s goals, feel content in their current work environment, and have no intention of leaving (Coetzee and Stoltz, 2015). Research confirms a significant positive correlation between high job satisfaction, organizational commitment, and retention intention (Soler, 2000). Job satisfaction stands out as a key factor influencing care workers’ retention intention in their positions (Cohen-Mansfield, 1997; Castle and Engberg, 2005). Ingersoll et al. (2002) discovered a strong positive correlation between job satisfaction and intention to stay (Ingersoll et al., 2002). Higher satisfaction with salary, job level, interpersonal relationships, and responsibilities significantly increased the likelihood of retention. Parsons et al. (2003) found that married care workers or those living with family members typically show a greater willingness to remain in their positions (Parsons et al., 2003). Castle et al. (2007) noted that increases in salary and job satisfaction not only lead to improved performance in care work but also result in higher retention rates within the original organization (Castle et al., 2007). Previous research consistently demonstrates a strong link between job satisfaction and employees’ intention to stay in their current positions.

### 2.5 Research hypotheses

Based on our literature review, we formulated the following hypotheses for Taiwanese home-care workers:

H1. Personal characteristics influence emotional labor, job satisfaction, and retention intention.

H1.1: Significant differences exist in emotional labor across various personal characteristics.

H1.2: Significant differences exist in job satisfaction across various personal characteristics.

H1.3: Significant differences exist in retention intention across various personal characteristics.

#### 2.5.1 Correlations exist between emotional labor, job satisfaction, and retention intention

H2.1: Significant correlations exist between the sub-dimensions of emotional labor and job satisfaction.

H2.2: Significant correlations exist between the sub-dimensions of emotional labor and retention intention.

H2.3: Significant correlations exist between the sub-dimensions of job satisfaction and retention intention.

#### 2.5.2 Cluster analysis

H3.1: Home care workers can be categorized into distinct groups based on their levels of job satisfaction, intention to remain in their position, and emotional labor practices.

H4: Examination of the Relationships among emotional labor, job satisfaction, and retention intention.

H4.1: Emotional labor is associated with job satisfaction.

H4.2: Emotional labor is associated with retention intention.

H4.3: Job satisfaction is associated with retention intention.

H4.4: Job satisfaction acts as a mediator in the relationship between emotional labor and retention intention.

This study aims to investigate the relationships between emotional labor, job satisfaction, and retention among home care workers in Taiwan. We hypothesize that emotional labor may impact job satisfaction and retention intention. Our findings seek to offer valuable insights for managers in hospitals and long-term care facilities to enhance their recruitment strategies and personnel management practices.

## 3 Methods

### 3.1 Study design and participants

**Figure 1:**
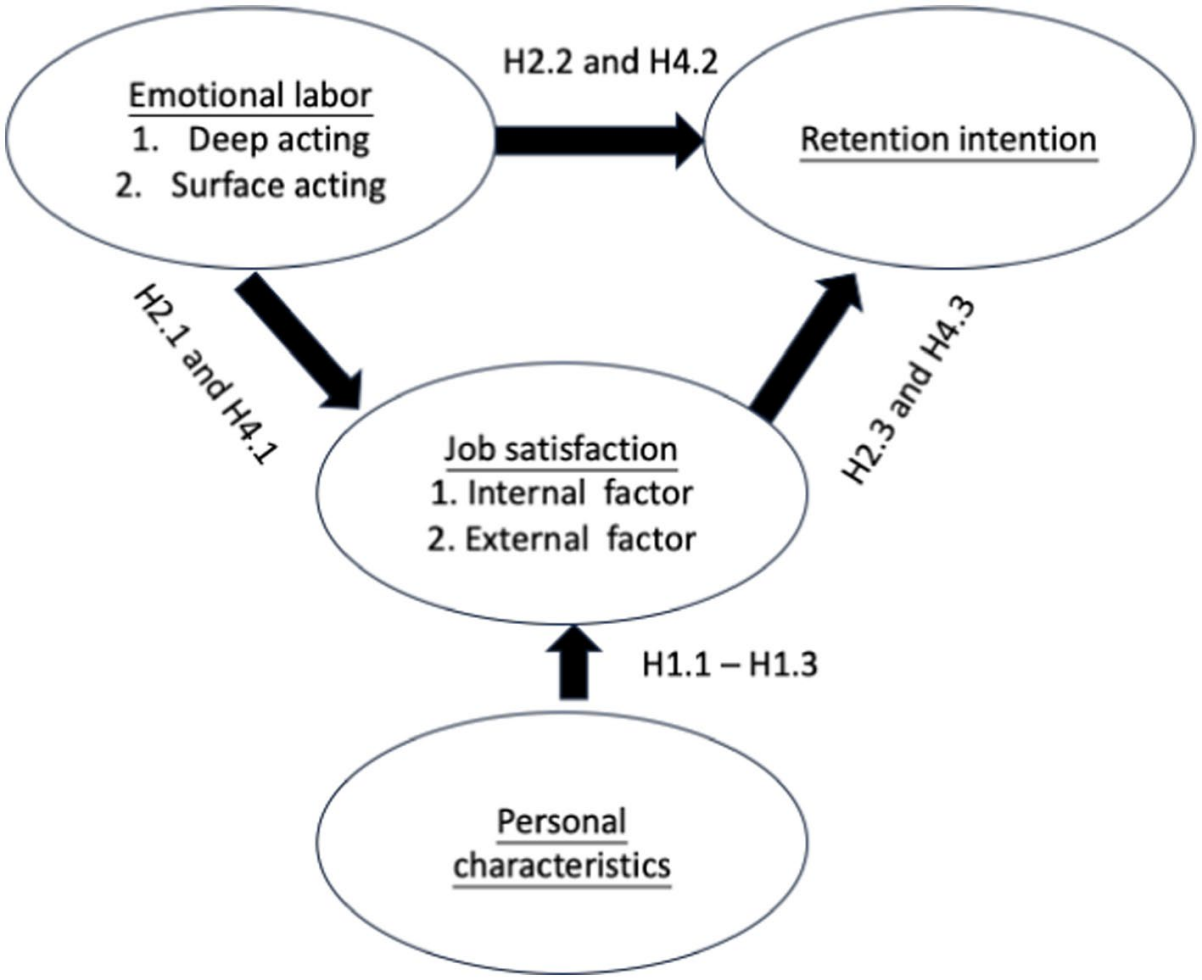
Research framework diagram.

This study has received approval from the Institutional Review Board of Ditmanson Medical Foundation Chia-Yi Christian Hospital, Taiwan (IRB Number: IRB2022050). This is a cross-section study design. The data were collected from home care workers in the Chiayi region of Taiwan. The inclusion criteria were as follows: (1) Home care workers aged 18 years or older, (2) Participants working in the Chiayi region of Taiwan, and (3) Participants who had worked in this position for more than 6 months. The exclusion criteria were as follows: (1) Participants unable to read Chinese, (2) Participants younger than 18 years old, and (3) Participants in leadership positions. We propose the following research framework (Figure 1) and four hypotheses. We calculated the required sample size using G*Power software, based on parameters for 1-way ANOVA: effect size of 0.25, power of 0.80, type I error of 0.05, and 4 groups. The initial calculation indicated a need for 269 participants.

### 3.2 Questionnaire survey

The questionnaire comprises respondents’ key characteristics and three variables: emotional labor, job satisfaction, and retention intention. Personal characteristics include gender, age, marital status, education level, years of caregiving experience, total monthly working hours, and average monthly salary. The emotional labor assessment consists of nine items based on Diefendorff et al. (2005) and Groth et al. (2009) covering surface acting (4 items) and deep acting (5 items) (Diefendorff et al., 2005; Groth et al., 2009). Job satisfaction assessment includes 16 items derived from Weiss et al. (1967) and Herzberg et al. (1959), addressing intrinsic satisfaction (8 items) and extrinsic satisfaction (8 items) factors (see Supplementary Table S1) (Herzberg et al., 1959; Weiss et al., 1967). Retention intention assessment features three items based on Ribelin (2003) (Ribelin, 2003) and Shahid (2018) (Shahid, 2018). All items use a 5-point Likert scale, ranging from 1 (“strongly disagree”) to 5 (“strongly agree”).

### 3.3 Statistical analysis

We used IBM SPSS Statistics software (version 28.0; SPSS, Chicago, IL, USA) for data analysis. Our methods included descriptive statistics, factor analysis, reliability analysis, and independent samples t-tests for comparing two groups. For comparisons involving more than two groups, we conducted one-way ANOVA followed by Schefft’s post-hoc test. We used Cronbach’s α to assess the internal consistency of each item. Pearson correlation coefficients estimated linear relationships between variables. We performed K-means cluster analysis to create two clusters, helping us understand subgroup characteristics and differences in retention intention. Path analysis, primarily through multiple regression, verified causal relationships among variables, with path coefficients indicating relationship strength. A *p*-value less than 0.05 was considered statistically significant.

## 4 Results

### 4.1 Sample structure descriptive statistical analysis

The study included 365 participants, with females comprising the majority (80.3%). Participants’ ages ranged from 18 to 60 years old, with the largest group being 51–55 years old (15.9%). The next most participant age groups were 56–60 (14.2%), 31–35 (13.4%), 36–40 (12.9%), 46–50 (11.8%), and 41–45 (10.1%). Younger age groups were less represented, with 21–25 years old at 7.1%, 26–30 at 6.8%, and the smallest group being 18–20 years old (3.0%). Among the participants, the vast majority were married (250, 68.5%), followed by single (81, 22.2%), divorced (26, 7.1%), and widowed (8, 2.2%). Regarding education level, most respondents had a college degree (43.6%). In terms of work experience, the majority had less than 5 years (216, 59.2%). For average monthly working hours, most worked between 141 to 180 h (357, 70.4%). As for average monthly salary, 333 participants (91.2%) reported earning between NT$25,000 and NT$45,000 (Tables 1, 2).

**Table 1:** Major characteristics of the study participants.

Variable		*n*	Percentage
Gender	Male	72	19.7
	Female	293	80.3
Age (year)	18 ~ 20	11	3
	21 ~ 25	26	7.1
	26 ~ 30	25	6.8
	31 ~ 35	49	13.4
	36 ~ 40	47	12.9
	41 ~ 45	37	10.1
	46 ~ −50	43	11.8
	51 ~ 55	58	15.9
	56 ~ 60	52	14.2
	61 ~ 65	17	4.7
Marital status	Married	250	68.5
	Unmarried	81	22.2
	Divorce	26	7.1
	Widowed	8	2.2
Education level	Junior high school or below	24	6.6
	Senior high school or vocational High school	113	31
	Junior college	159	43.6
	College	37	10.1
	Master or above	32	8.8
Service period (year)	≤5	216	59.2
	5–10	73	20
	11–15	51	14
	16–20	25	6.8
Total monthly working hours	61–80	11	3
	81–100	9	2.5
	101–120	28	7.7
	121–140	33	9
	141–160	112	30.7
	161–180	145	39.7
	>180	27	7.4
Average monthly income (New Taiwan Dollar)	≤25,000	15	4.1
	25,001–35,000	192	52.6
	35,001–45,000	141	38.6
	45,001–55,000	14	3.8
	55,001–65,000	3	0.8

**Table 2:** Measuring the reliability of emotional labor, job satisfaction, and retention intention.

Variable	KMO value	Bartlett’s test of sphericity			Cumulative explained variance (%)
		Approximately chi-square distribution	Degree of freedom	*p*-value	
Emotional labor	0.844	1463.16	36	0.001	63.177
Job satisfaction	0.916	4245.179	120	0.001	64.315
Retention intention	0.719	778.421	3	0.001	84.096

### 4.2 Factor analysis

Factor analysis was conducted on three variables: emotional labor (9 items), job satisfaction (16 items), and intention to stay (3 items). Table 3 summarizes the analysis results, including KMO values and Bartlett’s test of sphericity. The KMO values were 0.844 for emotional labor, 0.916 for job satisfaction, and 0.719 for intention to stay. These values surpass the 0.7 threshold, indicating strong inter-variable correlations.

**Table 3:** Validity analysis of emotional labor, job satisfaction, and retention intention.

Dimension	Sub-dimension	Number of items	Cronbach’s α	Cronbach’s α
Job satisfaction	External factor	8	0.915	0.933
	Internal factor	8	0.92	
Emotional labor	Deep acting	5	0.849	0.863
	Surface acting	4	0.791	
Retention intention		3	0.901	0.901

Bartlett’s test yielded a significance level of *p* < 0.001, confirming that the correlation matrix is not an identity matrix—thus suitable for factor extraction. Kaiser suggests that a KMO value exceeding 0.7 is appropriate for factor analysis (Kaiser and Rice, 1974). These results demonstrate that the questionnaire used in this study is well-suited for this analytical approach.

#### 4.2.1 Factor analysis of emotional labor

Table 4 details the factor analysis of the nine emotional labor items. For deep acting, factor loadings range from 0.695 to 0.813, with a characteristic value of 4.357, explaining 48.413% of the variance. Surface acting factor loadings span 0.516 to 0.864, with a characteristic value of 1.329, accounting for 14.765% of the variance. The total explained variance for emotional labor is 63.177%. These results indicate that both deep and surface acting are crucial components of emotional labor, with deep acting making a more substantial contribution to the overall construct.

**Table 4:** Correlation analysis among emotional labor, job satisfaction, and retention intention.

ID	1	2	3	4	5	6	7
Between Variables and Subdimensions	Emotional labor	Deep acting	Surface acting	Job satisfaction	External factor	Internal factor	Retention intention
Emotional labor	1						
Deep acting	0.901***	1					
Surface acting	0.850***	0.538***	1				
Job satisfaction	0.157**	0.142**	0.132*	1			
External factor	0.135**	0.110*	0.129*	0.912***	1		
Internal factor	0.146**	0.148**	0.104*	0.870***	0.591***	1	
Retention intention	0.115*	0.126*	0.07	0.048	0.062	0.02	1

*p-value<0.05; **p-value<0.01; ***p-value<0.001; *N*: not significant.

#### 4.2.2 Factor analysis of job satisfaction

A factor analysis was conducted on the 16 job satisfaction items, and the results are shown in Table 5. For external satisfaction, factor loadings ranged from 0.607 to 0.827, with an eigenvalue of 8.102, explaining 50.636% of the variance. For internal satisfaction, factor loadings spanned 0.629 to 0.891, with an eigenvalue of 2.189, accounting for 13.679% of the variance. The cumulative explained variance was 64.315%.

**Table 5:** Cluster analysis among home care workers.

Dimension	Cluster 1 (*n* = 235)		Cluster 2 (*n* = 130)		*t* value	*p*-value	Scheffe
	mean	SD	mean	SD			
Emotional labor	4.47	0.335	4.06	0.449	9.893	0.001***	1 > 2
Deep acting	4.52	0.372	4.08	0.524	9.279	0.002**	1 > 2
Surface acting	4.41	0.447	4.04	0.481	7.298	0.384	
Job satisfaction	4.48	0.293	3.76	0.344	20.266	0.058	
External factor	4.46	0.368	3.62	0.469	18.831	0.001***	1 > 2
Internal factor	4.5	0.372	3.89	0.373	14.99	0.040*	1 > 2
Retention intention	4.12	0.645	4	0.663	1.655	0.815	

*p-value<0.05; **p-value<0.01; ***p-value<0.001; *N*: not significant.

### 4.3 Reliability analysis

According to Xu and Fan (2023), a Cronbach’s α value above 0.70 indicates exhibit strong reliability (Xu and Fan, 2023). Our results, as presented in Table 3, show Cronbach’s α values exceeding 0.7 for all dimensions as well as their corresponding sub-dimensions. This demonstrates strong internal stability and consistency, affirming the reliability of our measurements.

### 4.4 Impact of major characteristics on all dimensions

#### 4.4.1 Gender

This study examined gender differences among home care workers across various dimensions, with results summarized in Supplementary Table S4. No significant differences were found in emotional labor or its sub-dimensions (surface acting and deep acting) between genders. Female workers reported higher average job satisfaction scores (4.26) compared to males (4.09). This pattern held true for both external satisfaction (females: 4.20, males: 4.00) and internal satisfaction (females: 4.31, males: 4.17). All these differences were statistically significant. No significant gender differences were observed in workers’ intention to remain in their current positions. Consequently, hypotheses H1.1 to H1.3 are partially supported by these findings.

#### 4.4.2 Age

Our analysis, presented in Supplementary Table S5, demonstrates variations among home care service workers across different age groups. No significant differences were found in emotional labor or its sub-dimensions (deep acting and surface acting) among age groups. Significant differences were observed in overall job satisfaction (*p* < 0.05) and both sub-dimensions: external satisfaction (*p* < 0.01) and internal satisfaction (*p* < 0.01). Post-hoc comparisons showed that home care service workers aged 46–50 and 51–55 reported significantly higher levels of internal satisfaction than those aged 26–30 (*p* < 0.01). No significant differences in retention intention were found across age groups. These results partially support hypotheses H1.1 to H1.3.

#### 4.4.3 Marital status

This study’s findings, presented in Supplementary Table S6, reveal differences in various dimensions among home care service workers based on their marital status. Significant differences were observed in overall emotional labor (*p* < 0.05) and its sub-dimension of surface acting (*p* < 0.05). However, no significant differences were found in the deep acting sub-dimension. However, post-hoc comparisons revealed no significant differences among marital status groups. For job satisfaction, significant differences were found in job satisfaction (*p* < 0.05) and its sub-dimension of external satisfaction (*p* < 0.01). Post-hoc comparisons indicated married workers showed higher job satisfaction than widowed workers (*p* < 0.05). Post-hoc comparisons indicated that married home care workers reported higher external satisfaction than unmarried workers or widowed workers (*p* < 0.01). A significant difference in intention to stay (*p* < 0.05) was found among marital status groups. However, post-hoc comparisons did not reveal any specific differences between these groups. These findings partially support hypotheses H1.1 to H1.3.

#### 4.4.4 Education level

This study examines differences among home care service workers across various educational levels, as presented in Supplementary Table S7. The analysis reveals no significant variations in emotional labor across different education levels. While job satisfaction and internal satisfaction showed no significant differences across education levels, external satisfaction varied significantly (*p* < 0.01). Post-hoc comparisons revealed that workers with junior college education reported higher external satisfaction than those with a college degree. For intention to stay, significant differences were observed among different education level. Post-hoc analyses revealed that workers with a senior high school or vocational high school degree had a higher intention to stay compared to those with a university degree. These findings partially support hypotheses H1.1 to H1.3.

#### 4.4.5 Length of service

Supplementary Table S8 summarizes the effect of service duration on the measured dimensions. The key findings indicate that emotional labor and retention intention did not differ significantly across groups with varying lengths of service. Job satisfaction (*p* < 0.01), external satisfaction (*p* < 0.05), and internal satisfaction (*p* < 0.01) showed significant differences across groups with varying lengths of service. Post-hoc comparisons revealed that workers with 6–10 years of service reported higher job satisfaction than those with less than 5 years of experience. In summary, home care service workers with 6–10 years of experience reported the highest overall job satisfaction across all service duration groups. In contrast, those with less than 5 years of experience exhibited the lowest levels of job satisfaction. These findings lend partial support to hypotheses H1.1 through H1.3.

#### 4.4.6 Monthly working hours

Supplementary Table S9 presents an analysis of differences among home care service workers based on their monthly working hours. No significant differences in overall emotional labor and deep acting, but a significant difference in surface acting (*p* < 0.01). Post-hoc comparisons reveal that worker in the 141–160 h and 161–180 h groups tend to conceal their true emotions more than those working 181 h or more. Significant differences were observed in job satisfaction (*p* < 0.001), external satisfaction (*p* < 0.001), and internal satisfaction (*p* < 0.05). Post-hoc comparisons indicate that workers in the 161–180 h group report higher satisfaction levels than those working 181 h or more. However, no significant differences in retention intention across different monthly working hour groups. These findings partially support hypotheses H1.1 to H1.3.

#### 4.4.7 Average monthly salary income

Supplementary Table S10 presents an analysis of differences among home care workers based on their average monthly salary. The findings reveal:

1. No significant differences in emotional labor and deep acting, but significant differences in surface acting (*p* < 0.05).
2. Significant differences in overall job satisfaction (*p* < 0.01) and external satisfaction (*p* < 0.001), but no significant difference in internal satisfaction. Post-hoc comparisons showed that home care workers earning 25,001–35,000 NTD (Group 2) and 35,001–45,000 NTD (Group 3) reported significantly higher external satisfaction than those earning below 25,000 NTD (Group 1).
3. No significant differences in retention intention.

The study indicates that home care workers earning 25,001–35,000 NTD monthly are more likely to conceal their true emotions at work. Those earning 25,001–45,000 NTD reported higher overall job satisfaction compared to other income groups. Retention intention did not differ across salary levels. These findings partially support hypotheses H1.1–H1.3.

### 4.5 Association among all dimensions

Table 4 displays the correlations between dimensions and sub-dimensions. Significant positive correlations exist between emotional labor and job satisfaction (r = 0.157, *p* < 0.01), including its sub-dimensions: external satisfaction (r = 0.135, *p* < 0.01) and internal satisfaction (r = 0.146, *p* < 0.01). Emotional labor also correlates positively with willingness to stay (r = 0.115, *p* < 0.05). Among emotional labor’s sub-dimensions, deep acting shows significant positive correlations with job satisfaction (r = 0.142, *p* < 0.01), external satisfaction (r = 0.110, *p* < 0.05), and internal satisfaction (r = 0.148, p < 0.01). Deep acting also correlates positively with willingness to stay (r = 0.126, *p* < 0.05). Surface acting exhibits significant positive correlations with job satisfaction (r = 0.132, *p* < 0.05) and its two sub-dimensions: external satisfaction (r = 0.129, *p* < 0.05) and internal satisfaction (r = 0.104, *p* < 0.05). However, surface acting does not significantly correlate with willingness to stay. Job satisfaction and its sub-dimensions—external and internal satisfaction—show no significant correlations with retention intention. These findings support hypothesis H2.1, partially support H2.2, and do not support H2.3.

### 4.6 Cluster analysis of home-care workers

The participants were arbitrarily classified into two clusters based on cluster analysis. Emotional labor (*p* = 0.001) and its sub-dimension of deep acting (*p* = 0.002) showed significant differences between the two clusters (Table 5). However, no significant difference was found in surface acting (*p* = 0.384). Job satisfaction did not show a significant difference between the two clusters. However, its external factor (*p* = 0.001) and internal factor (*p* = 0.040) showed significant differences between the clusters. Cluster 1 exhibited more genuine emotional expressions (deep acting) during work, demonstrated higher job satisfaction, and showed a greater willingness to stay. These findings suggest a positive correlation between authentic emotional engagement, job satisfaction, and retention intention. These results partially support hypothesis H3.1.

### 4.7 Path coefficients analysis

**Figure 2:**
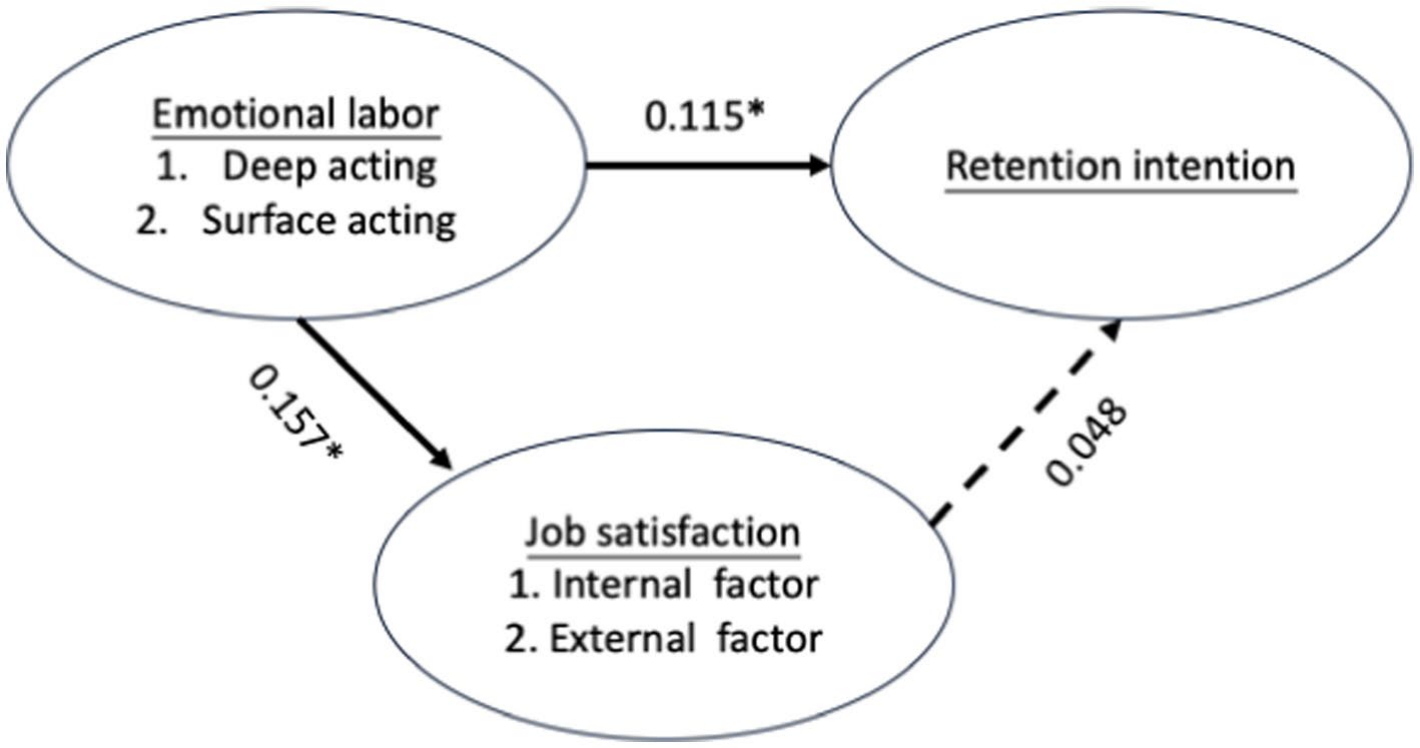
Results of path coefficients analysis. *: statistically significant.

Figure 2 and Table 6 present the path analysis results. Emotional labor exhibits a significant positive causal relationship with job satisfaction (β = 0.157, *p* < 0.01) and retention intention (β = 0.115, *p* < 0.05). However, job satisfaction shows no significant causal relationship with willingness to stay (β = 0.48, *p* > 0.05). These findings indicate that job satisfaction does not mediate the relationship between emotional labor and willingness to stay. Consequently, hypotheses H4.1, H2.2, H4.3, and H4.4 are not supported by these results.

**Table 6:** Path analysis of emotional labor, job satisfaction, and retention intention.

Path relationship	B	S.E	*t*	β	*R* ^2^
Emotional labor ➔ Job satisfaction	0.171	0.057	3.027**	0.157	0.025
Emotional labor ➔ Retention intention	0.176	0.08	2.200*	0.115	0.013
Job satisfaction ➔ Retention intention	0.067	0.073	0.919	0.048	0.002
Emotional labor ➔ Job satisfaction ➔ Retention intention	0.043	0.074	0.586	0.031	0.014

^*^B is the unstandardized regression coefficient; S.E. is the standard error; β is the standardized regression coefficient.

## 5 Discussion

Our study of Taiwanese home care workers revealed several key findings. While emotional labor showed no gender differences, female workers reported higher job satisfaction and willingness to stay. Job satisfaction varied significantly by age, with the 36–40 age group reporting the highest satisfaction, followed by those aged 41–45 and 51–55. The 26–30 age group reported the lowest satisfaction. Regarding marital status, divorced workers showed higher surface acting, while married workers reported higher overall job satisfaction. College graduates indicated higher external satisfaction with management, benefits, salary, case stability, and work flexibility compared to those with lower education levels. Experience played a role, as workers with 6–10 years of service reported higher satisfaction than those with less than 5 years. Monthly working hours affected behavior patterns—workers with 141–160 monthly hours were more likely to engage in surface acting, while those working 161–180 h reported the highest job satisfaction. Regarding income, workers earning 25,001–35,000 NTD showed higher surface acting, while those earning 25,001–45,000 NTD reported higher job satisfaction. The study found that emotional labor and deep acting both positively correlate with job satisfaction and willingness to stay. Interestingly, job satisfaction itself did not significantly correlate with willingness to stay.

Studies examining the relationship between age and job satisfaction show varying results. Some research indicates a positive linear correlation (Lee and Wilbur, 1985), while others find a U-shaped pattern (Clark et al., 1996). While age alone is not a strong predictor of job satisfaction, it interacts with other factors like job characteristics and personal evaluations (Bernal et al., 1998). Job characteristics and core self-evaluations play important roles, with their impact varying across age groups (Besen et al., 2013). Research shows that younger workers generally report lower satisfaction, especially with intrinsic job aspects, while older employees tend to be more satisfied with extrinsic factors (Lee and Wilbur, 1985). The U-shaped pattern suggests that job satisfaction typically decreases in early career stages before gradually rising towards retirement (Clark et al., 1996). This pattern helps explain our observation of significant age-related variations in job satisfaction.

Studies show that marital status significantly impacts how workers handle emotional labor and their job satisfaction. Research from Indonesia reveals that married workers face stronger negative effects from emotional and physical job demands compared to single workers (Elfira et al., 2020). When employees engage in surface acting—pretending to feel emotions they do not actually experience—it can cause exhaustion and anxiety that affects their marriages, leading to partner dissatisfaction and work–family conflicts (Krannitz et al., 2015). While our research found that married employees generally reported higher job satisfaction, there is currently no research examining how divorce affects surface acting at work.

Studies examining emotional labor’s impact on employee retention reveal complex relationships. Surface acting is associated with increased turnover intentions and emotional exhaustion, while deep acting shows mixed effects (Chau et al., 2009; Xu et al., 2017). Genuine emotions do not directly affect turnover intentions, but in-depth communication with colleagues moderates this relationship (Xu et al., 2017). Team climate plays a crucial role in mitigating the negative effects of emotional labor, particularly hiding emotions, on burnout and turnover intention (Cheng et al., 2013). Emotional labor engagement in work tasks can lead to higher retention rates by fostering greater workplace satisfaction and loyalty to the organization (Chau et al., 2009; Xu et al., 2017). However, some reports have shown that employees who engage in deep acting may be more likely to consider leaving their positions (Cho et al., 2017; Lim and Moon, 2023). In this study, emotional labor showed a positive correlation with retention intention, though job satisfaction did not act as a mediator (Table 6). However, our study did not examine the mixed effects of emotional labor on retention intention among Taiwanese home care workers.

The retention of qualified home care personnel represents a significant challenge in the LTC sector. Job satisfaction, encompassing both internal motivators and external rewards, plays a fundamental role in determining staff retention rates (Faul et al., 2010; Liu et al., 2020). Multiple elements influence employee retention, including demographic factors like age, level of education attained, compensation packages, and personal job satisfaction levels (Faul et al., 2010). Contrary to expectations, our study found no correlation between job satisfaction and retention intention in Taiwanese home care workers. However, we found that education level significantly affected retention intention, with college-educated workers showing the lowest retention intention. College-educated workers expect to have high-quality work opportunities. College graduates experience fewer “low quality” jobs compared to less-skilled workers (Bucheli, 2000). Therefore, they may not intend to stay in home care jobs for long periods.

This study has several limitations. While we established relationships between emotional labor, job satisfaction, and willingness to stay, we did not collect data on supervisory leadership styles. Consequently, we cannot determine whether these styles mediate the relationship between job satisfaction and willingness to stay. Furthermore, our study’s focus on home care workers in the Chiayi region of Taiwan may limit the generalizability of our findings to the broader Taiwanese home care workforce. To address this, future research should include participants from various Taiwanese cities, providing a more comprehensive understanding of the factors influencing home care workers’ retention.

## 6 Conclusion

Our research revealed several interesting patterns in the home care workforce. We found that while workers who engaged in emotional labor and deep acting tended to have higher job satisfaction and were more likely to stay in their positions, surprisingly, job satisfaction alone wasn’t a reliable predictor of retention intention. An intriguing paradox emerged among college-educated workers, who despite reporting higher satisfaction with workplace factors like management, benefits, and flexibility, were actually the least likely to remain in their positions—possibly because they had higher career aspirations. Gender and experience also played important roles: female workers showed greater job satisfaction and retention intention, and the most satisfied age group was 36–40 years old. Experience mattered too, with workers having 6–10 years of service reporting higher satisfaction than their less experienced colleagues. When it came to working hours, those putting in 141–160 h monthly tended to engage more in surface acting, while peak job satisfaction was found among those working 161–180 h.

## Data Availability

The raw data supporting the conclusions of this article will be made available by the authors, without undue reservation.
