# Supplementary material for: Emotional labor, job satisfaction, and retention among home care workers in Taiwan: a comprehensive analysis
Source: Front Psychol. 2025 Mar 31;16:1545955. doi: 10.3389/fpsyg.2025.1545955 (PMC11994665; doi:10.3389/fpsyg.2025.1545955)
Supplement: Supplementary file 1 [file Table_1.docx]

Table S1. The questionnaire used in this study.

| **Emotional Labor** |  |
| --- | --- |
| **Surface acting** |  |
| SU1 | When I am caring for a client, I appear cheerful on the outside, but my true emotions are not expressed internally. |
| SU 2 | When I am at work, I do my best to overcome my negative emotions and serve the clients I care for with a kind and friendly attitude. |
| SU 3 | Even when I know that the client I am caring for is being unreasonable, I hide my true emotions and work to solve their problems. |
| SU 4 | Although I am quite tired, I still make every effort to maintain an appropriate emotional state. |
| **Deep acting** |  |
| DE 1 | If I must express a certain emotion in front of others (such as kindness or gentleness), I try to do so genuinely rather than pretending. However, I still express my true emotions when appropriate. |
| DE 2 | When I'm feeling down, I may temporarily forget my unhappiness for the sake of work, but I still cannot completely hide my true emotions. |
| DE 3 | I strive to cultivate empathy, but I also face the clients I care for with my true emotions. |
| DE 4 | I work hard to cultivate empathy, but I also confront the clients I care for with my genuine emotions. |
| DE 5 | I make an effort to understand the issues faced by the clients I care for and provide solutions to alleviate their dissatisfaction, but sometimes I also express feelings of impatience. |
| **Job Satisfaction** |  |
| **Internal factor** |  |
| IN 1 | My job gives me a sense of accomplishment. |
| IN2 | I can complete tasks assigned by my supervisor independently. |
| IN 3 | My supervisor recognizes my job performance. |
| IN 4 | I receive recognition from service recipients for my job performance. |
| IN 5 | I receive recognition from clients’ family members for my job performance. |
| IN 6 | I am competent in the service items required for the job. |
| IN 7 | I can arrive at the service recipient's home on time. |
| IN 8 | I can complete my work within the specified time and maintain good quality. |
| **External factor** |  |
| EX 1 | I am satisfied with the benefits and systems provided by the organization (such as labor insurance, health insurance, and bonuses). |
| EX 2 | I am satisfied with my supervisor's decision-making ability. |
| EX 3 | I am satisfied with my current hourly wage and salary. |
| EX 4 | I am satisfied with the safety of the work environment. |
| EX 5 | I am satisfied with the location and distance of the assigned cases. |
| EX 6 | I am satisfied with the flexibility of my work schedule. |
| EX 7 | My current case load is stable. |
| EX 8 | I am happy and satisfied with my current job. |
| **Retention Intention** |  |
| RI 1 | I am willing to continue working as a home-based caregiver in the current institution. |
| RI 2 | I am satisfied with my current job and willing to continue working in this field. |
| RI 3 | I believe that my values are closely aligned with those of the institution. |

Table S2. Factor analysis of emotional labor.

| Dimension | item | Factor loading | |
| --- | --- | --- | --- |
|  |  | Factor1 | Factor2 |
| Deep acting | DA 4 | 0.813 |  |
|  | DA 3 | 0.803 |  |
|  | DA 5 | 0.78 |  |
|  | DA 2 | 0.711 |  |
|  | DA 1 | 0.695 |  |
| Surface acting | SA 1 |  | 0.864 |
|  | SA 2 |  | 0.859 |
|  | SA 3 |  | 0.705 |
|  | SA 4 |  | 0.516 |
| Eigenvalue | | 4.357 | 1.329 |
| Explained variation | | 48.413 | 14.765 |
| Cumulative proportion of explained variance | | 48.413 | 63.177 |

Table S3. Factor analysis of job satisfaction.

| Dimension | Item | Factor loading | |
| --- | --- | --- | --- |
|  |  | Factor1 | Factor2 |
| External factor | EX 5 | 0.827 |  |
|  | EX 3 | 0.802 |  |
|  | EX 7 | 0.786 |  |
|  | EX 8 | 0.764 |  |
|  | EX 6 | 0.755 |  |
|  | EX 4 | 0.747 |  |
|  | EX 1 | 0.737 |  |
|  | EX 2 | 0.607 |  |
| Internal factor | IN 4 |  | 0.891 |
|  | IN 5 |  | 0.87 |
|  | IN 6 |  | 0.799 |
|  | IN 3 |  | 0.729 |
|  | IN 7 |  | 0.718 |
|  | IN 2 |  | 0.717 |
|  | IN 8 |  | 0.674 |
|  | IN 1 |  | 0.629 |
| Eigenvalue | | 8.102 | 2.189 |
| Explained variation | | 50.636 | 13.679 |
| Cumulative proportion of explained variance | | 50.636 | 64.315 |

Table S4. Emotional labor, job satisfaction, and retention intention between sexes.

| **Variable** | **Mean** | | **student t** |
| --- | --- | --- | --- |
|  | **Man (n=72)** | **Female (n=293)** |  |
| **Emotional labor** | 4.35 | 4.32 | 0.481 |
| Deep acting | 4.4 | 4.35 | 0.846 |
| Surface acting | 4.27 | 4.28 | -0.72 |
| **Job satisfaction** | 4.09 | 4.26 | -2.847** |
| External factor | 4 | 4.2 | -2.685** |
| Internal factor | 4.17 | 4.31 | -2.358* |
| **Retention intention** | 3.96 | 4.1 | -1.508 |
| *p-value<0.05；**p-value<0.01；***p-value<0.001 | | | |

Table S5. Emotional labor, job satisfaction, and retention intention among age groups.

|  | Mean | | | | | | | | | | F value | Scheffe |
| --- | --- | --- | --- | --- | --- | --- | --- | --- | --- | --- | --- | --- |
| ID | 1 | 2 | 3 | 4 | 5 | 6 | 7 | 8 | 9 | 10 |  |  |
| Age group | 18-20 | 21-25 | 26-30 | 31-35 | 36-40 | 41-45 | 46-50 | 51-55 | 56-60 | 61-65 |  |  |
|  | n=11 | n=26 | n=25 | n=49 | n=47 | n=37 | n=43 | n=58 | n=52 | n=17 |  |  |
| **Emotional labor** | 4.52 | 4.16 | 4.2 | 4.28 | 4.32 | 4.37 | 4.41 | 4.38 | 4.37 | 4.18 | 1.606 |  |
| Deep acting | 4.51 | 4.25 | 4.23 | 4.29 | 4.37 | 4.42 | 4.4 | 4.43 | 4.4 | 4.2 | 1.104 |  |
| Surface acting | 4.55 | 4.05 | 4.15 | 4.28 | 4.26 | 4.3 | 4.41 | 4.3 | 4.32 | 4.16 | 1.745 |  |
| **Job satisfaction** | 3.99 | 4.08 | 3.97 | 4.25 | 4.32 | 4.31 | 4.28 | 4.31 | 4.17 | 4.13 | 2.367* | N |
| External factor | 3.84 | 3.96 | 4 | 4.24 | 4.36 | 4.28 | 4.2 | 4.22 | 4.05 | 3.96 | 2.539** | N |
| Internal factor | 4.14 | 4.19 | 3.94 | 4.26 | 4.29 | 4.34 | 4.37 | 4.41 | 4.3 | 4.31 | 2.510** | 7>3 8>3 |
| **Retention intention** | 3.79 | 3.79 | 3.97 | 4.08 | 4.07 | 4.09 | 4.11 | 4.19 | 4.17 | 4.04 | 1.172 |  |
| *p-value<0.05；**p-value<0.01；***p-value<0.001; N: not significant | | | | | | | | | | | | |

Table S6. Emotional labor, job satisfaction, and retention intention among marital status.

|  | Mean | | | | F value | Schefft |
| --- | --- | --- | --- | --- | --- | --- |
| ID | 1 | 2 | 3 | 4 |  |  |
| Ｍmarital status | Married | Unmarried | Divorce | Widowed |  |  |
|  | n=250 | n=81 | n=26 | n=8 |  |  |
| **Emotional labor** | 4.35 | 4.23 | 4.45 | 4.14 | 2.875* | N |
| Deep acting | 4.38 | 4.29 | 4.49 | 4.15 | 1.78 |  |
| Surface acting | 4.32 | 4.15 | 4.4 | 4.13 | 3.033* | N |
| **Job satisfaction** | 4.26 | 4.14 | 4.23 | 3.9 | 2.907* | 1>4 |
| External factor | 4.23 | 4.01 | 4.1 | 3.73 | 4.807** | 1>2 1>4 |
| Internal factor | 4.3 | 4.26 | 4.35 | 4.06 | 0.882 |  |
| **Retention intention** | 4.11 | 3.91 | 4.23 | 4.25 | 2.630* | N |
| *p-value<0.05; ***p-value<0.01; ***p-value<0.001; N: not significant | | | | | | |

Table S7. Emotional labor, job satisfaction, and retention intention among education levels.

|  | Mean | | | | | F value | Post-hoc comparison |
| --- | --- | --- | --- | --- | --- | --- | --- |
| ID | 1 | 2 | 3 | 4 | 5 |  |  |
| Education level | Junior high school or below | Senior high school or vocational high school | Junior college | College | Master or above |  |  |
|  | n=24 | n=113 | n=159 | n=37 | n=32 |  |  |
| **Emotional labor** | 4.32 | 4.34 | 4.34 | 4.27 | 4.28 | 0.284 |  |
| Deep acting | 4.41 | 4.38 | 4.36 | 4.34 | 4.29 | 0.308 |  |
| Surface acting | 4.21 | 4.28 | 4.31 | 4.2 | 4.27 | 0.521 |  |
| **Job satisfaction** | 4.14 | 4.26 | 4.26 | 4.09 | 4.1 | 1.917 |  |
| External factor | 3.98 | 4.16 | 4.26 | 3.93 | 4.07 | 3.640** | 3>4 |
| Internal factor | 4.31 | 4.37 | 4.26 | 4.25 | 4.14 | 1.846 |  |
| **Retention intention** | 4.15 | 4.12 | 4.1 | 3.72 | 4.11 | 2.969* | 3>4 2>4 |
| *p-value<0.05; **p-value<0.01; ***p-value<0.001; N: not significant | | | | | | | |

Table S8. Emotional labor, job satisfaction, and retention intention among service periods.

|  | **Mean** | | | | **F value** | Post-hoc comparison |
| --- | --- | --- | --- | --- | --- | --- |
| ID | 1 | 2 | 3 | 4 |  |  |
| Years of Service | ≤ 5 | 6-10 | 11-15 | 16-20 |  |  |
|  | n=216 | n=73 | n=51 | n=25 |  |  |
| **Emotional labor** | 4.28 | 4.41 | 4.37 | 4.35 | 2.259 |  |
| Deep acting | 4.31 | 4.45 | 4.45 | 4.35 | 2.136 |  |
| Surface acting | 4.24 | 4.38 | 4.28 | 4.35 | 1.734 |  |
| **Job satisfaction** | 4.16 | 4.36 | 4.29 | 4.28 | 4.239** | 2>1 |
| External factor | 4.09 | 4.31 | 4.25 | 4.15 | 3.172* | 2>1 |
| Internal factor | 4.22 | 4.41 | 4.34 | 4.41 | 4.003** | 2>1 |
| Retention intention | 4.01 | 4.13 | 4.25 | 4.11 | 2.088 |  |
| *p-value<0.05; ***p-value<0.01; ***p-value<0.001; N: not significant | | | | | | |

Table S9. Emotional labor, job satisfaction, and retention intention among monthly working hours.

|  | Mean | | | | | | | F value | Post-hoc comparison |
| --- | --- | --- | --- | --- | --- | --- | --- | --- | --- |
| ID | 1 | 2 | 3 | 4 | 5 | 6 | 7 |  |  |
| Monthly Working Hours | 61-80 | 81-100 | 101-120 | 121-140 | 141-160 | 161-180 | >180 |  |  |
|  | n=11 | n=9 | n=28 | n=33 | n=112 | n=145 | n=27 |  |  |
| **Emotional labor** | 4.09 | 4.25 | 4.33 | 4.4 | 4.36 | 4.34 | 4.12 | 1.966 |  |
| Deep acting | 4.2 | 4.31 | 4.36 | 4.47 | 4.37 | 4.37 | 4.24 | 0.762 |  |
| Surface acting | 3.95 | 4.17 | 4.29 | 4.31 | 4.35 | 4.31 | 3.97 | 3.226** | 5>7=6>7 |
| **Job satisfaction** | 3.99 | 4.07 | 4.18 | 4.26 | 4.21 | 4.32 | 3.91 | 3.909*** | 6>7 |
| External factor | 3.83 | 3.88 | 4.02 | 4.1 | 4.21 | 4.29 | 3.71 | 5.990*** | 5>7=6>7 |
| Internal factor | 4.16 | 4.26 | 4.35 | 4.41 | 4.2 | 4.35 | 4.12 | 2.221* | N |
| Retention intention | 3.72 | 4.15 | 4.23 | 3.88 | 4.09 | 4.12 | 3.96 | 1.56 |  |
| *p-value<0.05; **p-value<0.01; ***p-value<0.001; N: not significant | | | | | | | | | |

Table S10. Emotional labor, job satisfaction, and retention intention among average monthly salary.

|  | Mean | | | | | F value | Schiffer's test |
| --- | --- | --- | --- | --- | --- | --- | --- |
| ID | 1 | 2 | 3 | 4 | 5 |  |  |
| Average Monthly Salary Income (NT) | Below 2,500 | 25001-35000 | 35001-45000 | 45001-55000 | 55001-65000 |  |  |
|  | n=15 | n=192 | n=141 | n=14 | n=3 |  |  |
| **Emotional labor** | 4.11 | 4.35 | 4.33 | 4.2 | 4.04 | 1.833 |  |
| Deep acting | 4.23 | 4.39 | 4.36 | 4.24 | 4.13 | 0.82 |  |
| Surface acting | 3.97 | 4.31 | 4.29 | 4.14 | 3.92 | 2.469* | N |
| **Job satisfaction** | 3.9 | 4.25 | 4.25 | 4 | 3.81 | 3.485** | N |
| External factor | 3.7 | 4.21 | 4.2 | 3.74 | 3.67 | 5.564*** | 2>1 3>1 |
| Internal factor | 4.1 | 4.29 | 4.3 | 4.25 | 3.96 | 0.953 |  |
| **Retention intention** | 4.11 | 4.02 | 4.16 | 4.05 | 3.67 | 1.203 |  |
| *p-value<0.05; **p-value<0.01; ***p-value<0.001; N: not significant; NT: new Taiwan dollar | | | | | | | |
